# Emerging Roles of BAI Adhesion-GPCRs in Synapse Development and Plasticity

**DOI:** 10.1155/2016/8301737

**Published:** 2016-01-04

**Authors:** Joseph G. Duman, Yen-Kuei Tu, Kimberley F. Tolias

**Affiliations:** ^1^Department of Neuroscience, Baylor College of Medicine, One Baylor Plaza, Houston, TX 77030, USA; ^2^Integrative Molecular and Biomedical Sciences Program, Baylor College of Medicine, One Baylor Plaza, Houston, TX 77030, USA; ^3^Verna and Marrs McLean Department of Biochemistry and Molecular Biology, Baylor College of Medicine, One Baylor Plaza, Houston, TX 77030, USA

## Abstract

Synapses mediate communication between neurons and enable the brain to change in response to experience, which is essential for learning and memory. The sites of most excitatory synapses in the brain, dendritic spines, undergo rapid remodeling that is important for neural circuit formation and synaptic plasticity. Abnormalities in synapse and spine formation and plasticity are associated with a broad range of brain disorders, including intellectual disabilities, autism spectrum disorders (ASD), and schizophrenia. Thus, elucidating the mechanisms that regulate these neuronal processes is critical for understanding brain function and disease. The brain-specific angiogenesis inhibitor (BAI) subfamily of adhesion G-protein-coupled receptors (adhesion-GPCRs) has recently emerged as central regulators of synapse development and plasticity. In this review, we will summarize the current knowledge regarding the roles of BAIs at synapses, highlighting their regulation, downstream signaling, and physiological functions, while noting the roles of other adhesion-GPCRs at synapses. We will also discuss the relevance of BAIs in various neurological and psychiatric disorders and consider their potential importance as pharmacological targets in the treatment of these diseases.

## 1. Introduction

Mental, emotional, and autonomic functions of the brain arise from interactions between the nearly 100 billion neurons that comprise this organ in humans. On average, each neuron forms 1,000 specialized contacts, or synapses, with other neurons. Synapses are asymmetric, complex, and highly dynamic [1, 2]. The plasticity of synapses and dendritic spines, the morphological structures that are the loci of most excitatory synapses in the central nervous system (CNS), are widely believed to underlie learning and memory and are frequently altered in neurodevelopmental and neurodegenerative diseases [3, 4]. Thus, understanding the development, dynamics, and elimination of synapses is crucial for human health. A dizzying array of signals coordinates these processes, and thus receptors are an integral component of the synaptic regulatory machinery [1–4]. Receptors also represent the most accessible point at which to manipulate these processes pharmacologically [5].

## 2. Adhesion-GPCRs

G-protein coupled receptors (GPCRs) comprise a superfamily of approximately 800 members in humans, including many important drug targets [6]. They exhibit a characteristic seven-transmembrane (7TM) core structure by which GPCRs interact with and activate a variety of heterotrimeric G-proteins, which in turn activate or repress intracellular signaling cascades [7]. Adhesion-GPCRs are a GPCR subfamily with 33 members in humans that are characterized by an extended N-terminal extracellular segment connected to the core GPCR structure by a distinctive GPCR autoproteolysis-inducing (GAIN) domain, which is present in all adhesion-GPCRs except GPR123 [8, 9]. The N-terminal segments of most adhesion-GPCRs contain multiple domains capable of binding to other cells or the extracellular matrix [8–10]. These include at least 16 different types of domain, with multiple types frequently occurring within the same protein; domains include cadherin-like repeats, thrombospondin-like repeats, rhamnose-binding lectin domains, and calnexin domains. Adhesion-GPCRs can be divided into 9 subfamilies based on phylogenetic analysis of the GPCR moiety; members of the different subfamilies generally also have related complements of N-terminal adhesive domains [9, 10]. GAIN domains mediate autoproteolytic cleavage of adhesion-GPCRs during translation in the ER at a site within the GAIN domain called the GPCR proteolysis site (GPS) [11, 12]. After cleavage, the N- and C-terminal fragments (NTFs, CTFs) of most adhesion-GPCRs remain noncovalently associated [9, 10]. However, this scenario is complicated. Some adhesion-GPCRs do not undergo autoproteolysis, and some that do may even swap NTFs with other adhesion-GPCRs resulting in “hybrid” adhesion-GPCRs [9, 13, 14]. Cell type and ligand binding may affect cleavage and association of the resulting fragments. It has been widely believed that the NTFs may repress the signaling mediated by CTFs, and that ligand binding relieves this inhibition, possibly by causing dissociation of the NTF from the CTF [8, 15]. Recently, a peptide agonist sequence named Stachel was identified on the C-terminal side of the GPS of adhesion-GPCRs. This sequence, which is specific for a given adhesion-GPCR, can activate G-protein dependent signaling through the adhesion-GPCR when it is unmasked by removal of the NTF or conformational changes in the protein (either of which is presumably ligand-induced) [16]. Identification of the GAIN domain and Stachel sequence are both recent findings, illustrating a rapid advance in the knowledge of adhesion-GPCR biology after years lagging behind other GPCRs.

Adhesion-GPCRs function in various tissues throughout organisms [8, 9], but an important driving force of recent rapid advances in adhesion-GPCR biology has been the discovery that adhesion-GPCRs regulate the development and function of many aspects of the nervous system. These include migration of neuronal precursors, axon guidance, myelination of axons, vascularization of the brain, and synapse formation and function [8, 9]. In this brief review, we highlight the roles of the brain-specific angiogenesis inhibitor (BAI) subfamily of adhesion-GPCRs at neuronal synapses. Adhesion-GPCR nomenclature arose over a long period of time and in a nonsystemic manner. Recently, a systemized nomenclature was proposed for this family [9]. Thus, the members of the BAI subfamily, BAI1–3, would now be named ADGRB1–3. This new nomenclature is not yet in standard use, and we will use the traditional names for adhesion-GPCRs, noting the new designations of adhesion-GPCRs we discuss. For general information on adhesion-GPCR function we direct the reader to several excellent recent reviews [8–10, 12].

## 3. The BAI Subfamily of Adhesion-GPCRs

**Figure 1:**
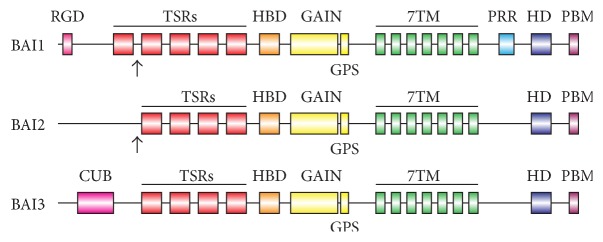
Schematic representation of BAI family members. BAI adhesion-GPCRs have a diverse collection of signaling and structural domains. These include thrombospondin type I repeats (TSRs), a hormone binding domain (HBD), the GAIN domain (GAIN), the GPCR autoproteolysis site (GPS), the characteristic seven-transmembrane domain (7TM), an *α*-helical RKR motif (HD), and the PDZ-binding motif (PBM), which are shared between all three family members. BAI1 has five TSRs, while BAI2 and BAI3 only have four. BAI1 and BAI2 are cleaved by proteases (BAI1 by matrix metalloprotease-14, BAI2 by Furin), which generates truncated fragments at the indicated locations marked by arrows. BAI1 has an additional integrin-binding RGD motif in the N-terminus and a proline-rich region (PRR) in the C-terminus. BAI1 also has a slightly truncated third intracellular loop compared to the other family members. BAI3 has a unique CUB domain in the N-terminus.

BAI1, BAI2, and BAI3 (ADGRB1–3) comprise a subfamily of adhesion-GPCRs that are highly expressed in the brain [9, 17]. BAIs are large proteins, approximately 200 kDa in size, with each possessing a long N-terminal region containing multiple adhesive thrombospondin type 1 repeats (TSRs), a hormone-binding domain, and the autoproteolysis-inducing GAIN domain (Figure 1). BAIs also contain an extended intracellular region C-terminal to the conserved 7TM GPCR domain that terminates in a PDZ-binding motif, QTEV (Gln-Thr-Glu-Val) [18]. BAI1 contains an additional TSR (five in total), an integrin-binding RGD (Arg-Gly-Asp) motif, and a C-terminal proline-rich region not present in the other two BAI family members (Figure 1).

*BAI1* was initially identified as a target gene of the tumor suppressor p53 [19]. Genes encoding BAI2 and BAI3 were subsequently discovered based on their homology with* BAI1* [20]. BAIs are widely expressed in postnatal and adult brain, with* BAI1* and* BAI2* mRNA levels peaking at postnatal day 10 (P10), while the level of* BAI3* mRNA is highest 1 day after birth [21]. BAI1 protein is present in neurons, glia, and macrophages, with particularly high expression in cortical and hippocampal pyramidal neurons [22–26]. Less is known about the cellular distribution of BAI2 and BAI3 proteins, although BAI3 is abundant in cerebellar Purkinje cells [27–29]. In neurons, BAI1 and BAI3 are both enriched in the postsynaptic density (PSD), suggesting a role for these proteins in synapse development and/or function [25, 30, 31].

Like most adhesion-GPCRs, BAIs possess a GAIN domain, but their ability to undergo autoproteolytic cleavage appears to be cell-type specific and not required for proper surface trafficking [31]. For instance, while BAI1 is cleaved at the GPS site in mouse brain and human malignant glioma cells [11, 32–34], uncleaved full-length BAI1 is also clearly present in hippocampal and cortical neurons [25]. Cleavage of the BAI1 GAIN domain generates a secreted 120 kDa fragment called Vasculostatin-120 (Vstat120), which is capable of inhibiting angiogenesis and tumor formation [32, 33]. BAI1 is also cleaved at a second site N-terminal to the GAIN domain by matrix metalloproteinase 14 (MMP-14) [35]. This cleavage event generates a 40 kDa fragment called Vasculostatin-40 (Vstat40), which also has antiangiogenic activity [35]. The antiangiogenic effects of Vstat120 and Vstat40 are primarily mediated by the TSRs, which bind to the scavenger receptor CD36 and induce proapoptotic signaling [33]. While proteolytic cleavage of BAI proteins is thought to both modulate the function of the full-length receptors and release their NTFs, which can exert their own physiological effects [18], more work needs to be done to understand how cleavage is regulated and what precise consequences it has on BAI function.

Research in the last decade has revealed a number of important roles for BAI family members in diverse cellular processes [17, 36]. As indicated above, BAI proteins can function as potent inhibitors of angiogenesis and tumor progression [36]. BAI1 expressed in macrophages has also been shown to bind to phosphatidylserine (PS) and lipopolysaccharide (LPS) and mediate the engulfment of apoptotic cells and Gram-negative bacteria, respectively [24, 37]. BAI1 promotes engulfment in response to PS or LPS binding by activating the associated ELMO/DOCK180 signaling module, which in turn activates the small GTPase Rac1 and induces Rac1-dependent actin cytoskeletal remodeling required for internalization of apoptotic cells or bacteria [24, 37]. The ability to BAI1 to bind to PS is also important for myoblast fusion, and loss of BAI1 results in a reduction in myofiber size and impaired muscle regeneration in mice [38]. The TSRs on the N-terminus of BAI family members are essential for their capacity to regulate these diverse cellular processes, and therefore proteolysis of the BAI extracellular domain may dramatically alter BAI function [36].

## 4. Roles of BAIs at Synapses

Despite the recent advances in our understanding of BAI function, until recently, little was known about the roles of BAI adhesion-GPCRs in neurons. Over the last few years, BAIs have emerged as important regulators of synaptogenesis and synaptic plasticity. Below, we consider the synaptic functions of each of the BAI family members in turn.

### 4.1. BAI1 Function at Synapses

BAI1 is enriched in, though not exclusively localized to, the PSD in dendritic spines in hippocampal neurons; this has been shown by biochemical fractionation and immunocytochemistry in rat hippocampal neurons and mouse brains [25, 31]. This enrichment indicated that BAI1 might play a role in synaptic formation or function, and this problem was attacked in two different ways. In both cases, synaptic effects were found, though the details vary.

**Figure 2:**
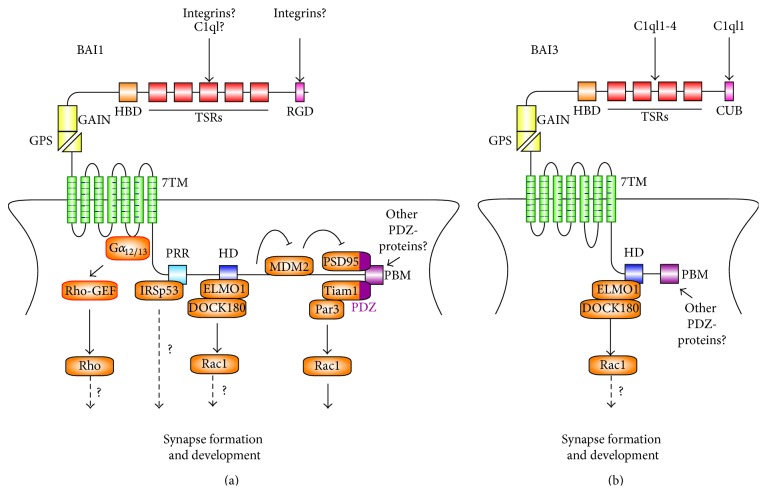
Synaptic binding partners and signaling pathways of BAI adhesion-GPCRs. (a) Synaptic interactions of BAI1. On the N-terminal segment of BAI1, the TSRs and the RGD motif are predicted to bind integrins. The TSRs also putatively bind complement C1ql factors, although the function of this interaction is unclear. BAI1 activates the RhoA pathway by coupling with G*α*_12/13_, although this has only been shown in cultured HEK293T cells and requires confirmation in neurons (red outline). The C-terminal region of BAI1 binds to IRSp53 via its proline-rich region (PRR), but the function of this interaction needs to be further explored. BAI1 also interacts with the Rac1 activator modules ELMO1/DOCK180 (via the *α*-helical RKR motif (HD)) and Tiam1/Par3 (via the PDZ-binding motif (PBM)). However, only the Tiam1/Par3 interaction is required for BAI1's effects on dendritic spine formation and excitatory synaptogenesis. In addition, BAI binds to the ubiquitin E3 ligase MDM2 and suppresses its polyubiquitination activity on PSD95, stabilizing PSD95 expression levels. (b) Synaptic interactions of BAI3. The TSRs and the CUB domain of BAI3 have been shown to bind complement C1ql factors C1ql3 and C1ql1, respectively. In cerebellar development, the C1ql1-BAI3 interaction helps establish proper synaptic connectivity in Purkinje cells and maintain a single-winner climbing fiber. The *α*-helical RKR motif (HD) of BAI3 also interacts with ELMO1/DOCK180 to regulate dendritogenesis, but the role of this interaction in synaptogenesis remains to be determined.

Our approach was to acutely knock down BAI1 both* in vitro* using cultured rat hippocampal neurons and* in vivo* using* in utero* electroporation of shRNAs directed against BAI1 [25]. In both systems, we found that BAI1 plays a key role in dendritic spine formation. Knockdown of BAI1 in cultured primary hippocampal neurons resulted in a loss of spine and synapse density with a shift of remaining spines to an immature elongated morphology [25].* In vivo* knockdown also resulted in a dramatic loss of spine density and a shift toward less mature spines in the somatosensory and the cingulate cortices [25]. BAI1's prospinogenic and prosynaptogenic activities are mediated through its interactions with the cell polarity complex Tiam1/Par3 through its C-terminal PDZ-binding motif [25] (Figure 2). Tiam1 is an activator of the small GTPase Rac1, which directs the actin cytoskeletal remodeling that drives spine and synapse development [39]. Tiam1 couples Rac1-dependent spine and synapse formation to extracellular signals, including glutamate (via NMDA receptors) [40], ephrin-B (via EphB receptors) [41], and BDNF (via TrkB receptors) [42]. BAI1 anchors the Tiam1/Par3 complex to dendritic spines where localized Rac1 activation promotes the formation of dendritic spines and subsequent excitatory synaptogenesis. Of note, although other Rac1 activators such as ELMO/DOCK180 bind to BAI1 [24], Rac1 activation leading to spinogenesis requires only Tiam1, as BAI1 mutants lacking the Tiam1/Par3-interacting motif cannot rescue the knockdown phenotype, whereas mutants that do not interact with ELMO/DOCK180 can [25].

Consistent with these results, knockout mouse studies recently revealed a requirement for BAI1 in spatial learning and synaptic plasticity [26]. BAI1-null mice have severe deficits in both hippocampus-dependent spatial learning and memory along with enhanced long-term potentiation (LTP) and impaired long-term depression (LTD) [26]. An interesting result arising from this study was the discovery that BAI1 contributes to proper synapse formation through its ability to stabilize the expression of the postsynaptic scaffold protein PSD95. BAI1-null mice show significant decreases in PSD95 at dendritic spines/synapses. It was determined that BAI1 binds to and inhibits the E3 ubiquitin ligase MDM2, thereby preventing the PSD95 degradation that was responsible for the spatial learning and plasticity phenotypes observed in BAI1-null mice [26] (Figure 2).

Although both of these studies agreed that BAI1 plays a role in synapse function, there were important differences in the results. Our results using shRNAs against BAI1 led to stark and obvious loss of spines, while the results with the BAI1-null mice showed no difference in spine density. There are obvious differences in the techniques used that could have given rise to these differences, and we will return to this issue below.

BAI1's C-terminal PDZ-binding motif also interacts with a variety of other synaptic molecules. Proteomic analysis reveals that the C-terminal segment of BAI1 can bind to PDZ-domain-containing proteins such as SAP97 (DLG1), Densin-180, MAGI-1/BAP1, MAGI-2, and MAGI-3 [31]. However, the exact functions of the majority of these interactions are not well understood. One potentially interesting BAI1-binding protein is the insulin receptor substrate 53 (IRSp53), which binds to a proline-rich region in BAI1's intracellular C-terminal segment and is also enriched in the PSD [43, 44]. Since IRSp53 is itself a downstream effector of Rac1 and Cdc42 and a regulator of dendrite spine morphogenesis [45], future studies that explore the effects of IRSp53-BAI1 interactions could elucidate key mechanisms of spinogenesis and synaptogenesis. IRSp53's potential role in autism spectrum disorder (ASD) makes this an even more interesting interaction to investigate [46].

### 4.2. BAI2 Function at Synapses

Like BAI1, BAI2 is broadly expressed in the brain, primarily in neurons and astrocytes [47]. However, the subcellular localization of BAI2 remains unclear. Roles for BAI2 in neurogenesis and synaptogenesis have been suggested but not well established experimentally. BAI2-deficient mice were found to display increased resistance to social defeat stress and reduced immobility in the tail suspension test, two behavioral assays that assess depressive behavior in rodents [48]. BAI2-deficient mice were also shown to exhibit increased neurogenesis in the dentate gyrus of the hippocampus, where BAI2 is highly expressed [47, 48]. These two observations are likely related since enhanced adult neurogenesis has been shown to positively correlate with resistance to depression [49]. It is also consistent with reports that BAI2 suppresses the expression of vascular endothelial growth factor (VEGF) [50], as VEGF stimulates adult neurogenesis in the dentate gyrus [51]. Loss of BAI2 could therefore increase VEGF levels, resulting in enhanced neurogenesis and increased resistance to stress. This idea will need to be further investigated. Furthermore, since stress and depression are known to induce synapse loss, while antidepressants promote synaptogenesis [52], in future studies it will be interesting to investigate the possible roles of BAI2 at synapses.

### 4.3. BAI3 Function at Synapses

Biochemical fractionation studies have revealed that like BAI1, BAI3 localizes to excitatory synapses in the brain [30, 53]. Furthermore, overexpression studies examining the localization of BAI3 in transfected hippocampal neurons have shown that it is highly enriched in spines where it colocalizes with the postsynaptic marker PSD95 [28]. Together these findings suggest that BAI3 may play an important role at excitatory synapses. Indeed, recently BAI3 was shown to regulate excitatory synapse connectivity and formation in the mouse cerebellum [28, 29] (Figure 2). Knockdown of BAI3 using lentivirus-delivered shRNA in P7 pups induced clear deficits in connectivity between cerebellar climbing fibers and their target Purkinje cells* and* between parallel fibers and Purkinje cells by P21 [28]. Dendritic spine density and vGlut1-positive synaptic contacts were both decreased in Purkinje cells with reduced BAI3 levels [28]. Similarly, mice lacking BAI3 specifically in Purkinje cells show a significant decrease in the number of vGlut2-positive puncta in the cerebellum [29].

BAI3's role at climbing fiber synapses is mediated through its interactions with a class of secreted complement proteins known as the C1q-like complement (C1ql) family. C1ql proteins are broadly expressed in the brain with different spatial and temporal expression patterns shown by family members C1ql1–4 [54]. In particular, C1ql1 is highly expressed during the first 2 postnatal weeks in various neuronal populations, particularly in the hippocampus, cerebral cortex, and cerebellum [54]. Transient C1ql1 secretion in the cerebellum promotes Purkinje cell spinogenesis, and the effect of modulating C1ql1 expression on Purkinje cell spinogenesis depends on the expression levels of BAI3 [28]. Critically, the C1ql1-BAI3 interaction promotes developmental synapse refinement and triggers elimination of surplus climbing fiber synapses, helping to select and maintain a single winning climbing fiber [29]. BAI3 expression in Purkinje cells is required for this process, and the climbing fiber is the source of C1ql. Moreover, continued expression of BAI3 is necessary for maintenance of climbing fiber synapses, and adult mice lacking C1ql, which possess excess climbing fiber synapses per Purkinje cell, eliminate these extra synapses when C1ql is introduced into the animals [29].

C1ql1 interacts with BAI3 through the N-terminal CUB domain, which is unique to BAI3 [29]. BAI3 also interacts with another C1ql family member, C1ql3, through its TSRs [55]. Incubating cultured hippocampal neurons with C1ql3 was shown to decrease excitatory synaptic density, and this effect was reversed by adding the isolated TSRs of BAI3 to the culture [55]. This result suggests a role for BAI3/C1ql3 in hippocampal synapse development akin to the BAI3/C1ql1-mediated pruning function in the cerebellum described above. It is not known if BAI3 also plays an earlier role in promoting synapse formation in the hippocampus. Further, since the TSRs in BAI3 are present in all BAIs, it is possible that C1ql3 also interacts with BAI1 and BAI2, but this remains to be investigated.

BAI3's role in synapse elimination during cerebellar development could shed some light on the differences observed in the shRNA-transfected versus BAI1-null mice described above. If proper spine formation requires a competition to sort out the “winning” synapse, expression profiles of relevant proteins in participating neurons might contribute to the resolution of this competition. In the neurons in which BAI1 was removed via shRNA, only the transfected cells had a deficit in BAI1, and they represented a small fraction (<5%) of the total population. If they were in competition with BAI1-expressing neurons for the establishment of synapses,* and* BAI1 promotes winning the competition, then the BAI1 knockdown neurons would be at a decided disadvantage relative to the vast majority of neurons expressing normal levels of BAI1. This state of affairs would hold for both the cultured neurons and the* in vivo* preparations. On the other hand, the neurons examined in the BAI1 null mice existed on a background of BAI1 null neurons. Therefore, the unmarked neurons would not have an advantage in preserving synapses and this may explain why no loss of dendritic spines was observed. Such argument by analogy can only go so far, and compensation by other BAI family members could also be a factor, but this hypothesis warrants further investigation.

## 5. Other Adhesion-GPCRs Involved in Synapses

In addition to the roles that BAIs play in synaptogenesis and synaptic function, there is evidence that additional adhesion-GPCRs function in these roles. Latrophilins are an adhesion-GPCR subfamily comprised of 3 members latrophilins 1–3 (Lphn1–3 or ADGRL1–3) and ELTD1 (ADGRL4) in humans and represent one of only two subfamilies conserved in invertebrates [9]. Latrophilins were identified as receptors for the black widow spider toxin *α*-latrotoxin, which causes a massive Ca^2+^-mediated exocytosis of neurotransmitter-containing vesicles from the presynaptic side of the synapses [56]. Lphn1 and Lphn3 are largely restricted to the brain, while Lphn2 is expressed in many tissues [9]. In addition to their GAIN domains, Lphns contain a hormone receptor motif, an olfactomedin-like domain, and a rhamnose-binding lectin domain in their NTFs [9]. Both Lphn1 and Lphn3 have been implicated in synapse formation. Lphn1 is thought to mediate its effects on synapse formation via interactions with teneurin-2/lasso [57, 58], neurexin-1*β*/2*β* [59], and fibronectin leucine-rich transmembrane proteins (FLRTs) [58]. All three of these proteins have been implicated independently in synapse formation. Presynaptic Lphn1 binds to teneurin-2 via its lectin domain with nanomolar affinity in a manner regulated by alternate splicing of Lphn1 [57, 58]. This interaction supports cell adhesion, while homophilic interaction between teneurins does not [58]. The Lphn1/teneurin interaction leads to presynaptic Ca^2+^ increases [57], and disruption of the interaction using the teneurin-binding segment of the Lphn1 NTF decreases both excitatory and inhibitory synapse density in rat hippocampal neurons [58]. Lphn1's interaction with neurexins also has nanomolar affinity and is regulated by alternate splicing of neurexins but is largely mediated by Lphn1's olfactomedin domain [59]. This interaction is especially intriguing because neurexins and their canonical binding partners, neuroligins, form trans-synaptic complexes and are strongly implicated in ASD [60]. Postsynaptic Lphn1 binds to presynaptic neurexins competitively with neuroligins [59]. It is not yet known what function the Lphn1/neurexin interaction serves at synapses, but given the known roles of both proteins, it is likely to be of high interest. Similarly, the role of the Lphn1/FLRT-3 interaction is not completely understood. Lphn3 has received increased attention of late due to a strong emerging correlation with attention deficit/hyperactivity disorder (ADHD) in humans [61, 62]. Lphn3 binds to FLRT-3 via its olfactomedin domain and to teneurin-1 via its olfactomedin and lectin domains [63, 64]. Presynaptic Lphn3 interacts with postsynaptic FLRT-3 to promote synapse formation in hippocampal neurons and in cortical synapses from layers 2/3 to layer 5 [63, 64]. Interestingly, FLRT-3 and teneurins vary in their distributions throughout the layered structure of the cortex, suggesting that Lphn3 could serve different functions in different regions of the brain by interacting with distinct ligands [64]. In short, Lphns are implicated in both presynaptic function and in directing synapse formation by forming complexes with transmembrane ligands in neuronal membranes.

The Celsr adhesion-GPCR subfamily is characterized by the presence of atypical cadherin repeats, calcium-binding EGF-like domains, laminin G domains, and a hormone receptor motif in their NTFs in addition to the GAIN domain [9, 10]. Like latrophilins, this subfamily is conserved in invertebrates, with Flamingo in* Drosophila melanogaster*, Fmi-1/2 in* Caenorhabditis elegans*, and Celsr1–3 (ADGRC1–3) in humans [9, 10]. Adhesion-GPCRs of the Flamingo/CELSR subfamily function in many aspects of nervous system development, including neural tube closure, axon guidance, and the formation of dendritic arbors [9, 65–68]. These effects are mediated through the now classical interaction of these proteins with the cellular planar cell polarity (PCP) machinery, as well as cAMP- and Ca^2+^-dependent mechanisms [9, 65, 66, 68]. Synaptic defects are observed when expression of Celsr-subfamily adhesion-GPCRs is altered or repressed, but it is difficult to determine whether these are direct effects on synaptic formation and/or maintenance, or whether they arise secondarily from malformation of axons and dendrites. Loss of Flamingo leads to formation of ectopic neuromuscular junctions, or synapses between axons and muscle, in Drosophila [69]. It also leads to malformed en passant synapses in this system, though these synapses are functional [69]. Further, aging animals lacking Flamingo exhibit a decrease in neuromuscular junctions, though this appears to be an effect of axonal degeneration [69]. Numerous questions remain to be answered in order to determine the specific roles of Celsr subfamily adhesion-GPCRs in synaptic formation and function. Finally, very large GPCR 1 (VLGR1 or ADGRV1) has been implicated in the formation of cochlear synapses, though its specific role remains unclear [70]. Many adhesion-GPCRs have not yet been tested for a role in synapses. Identification of adhesion-GPCRs involved in synaptic formation and function as well as elucidation of the mechanisms and signals that underlie these roles are important challenges for both adhesion-GPCR and synaptic biology.

## 6. BAIs' Disease Relevance and Potential as Therapeutic Targets

Given the important roles that BAI adhesion-GPCRs play in promoting synapse development and plasticity and inhibiting angiogenesis and tumor formation [18], it is not surprising that they have been implicated in a number of human diseases. For instance, single nucleotide polymorphisms (SNPs) and copy number variations in the human* BAI3* gene have been associated with schizophrenia [71–73], bipolar disorder [74], and drug addiction [75], brain disorders characterized by synapse abnormalities [4]. Furthermore, BAI3 expression is affected by lithium treatment, which is often used to treat patients with bipolar disorder and schizophrenia [74, 76]. The human* BAI1* gene is also located in a hot spot for* de novo* germline mutations in patients with autism [77], and BAI1 expression is upregulated in mouse models of Rett and MeCP2 Duplication Syndromes [78]. Conversely,* BAI1* expression is downregulated in glioblastoma and is inversely correlated with neovascularization in colorectal and lung cancers [36]. The growing evidence that BAIs play critical roles in human disease suggests that they may make good therapeutic targets in the future. GPCRs are generally considered to be the most successful therapeutic targets for a broad spectrum of diseases. Indeed, greater than 50% of the current therapeutic agents on the market target these proteins [79, 80]. Greater insight into the regulation and function of BAIs could therefore facilitate the development of novel therapies for the treatment of brain disorders and cancer.

## 7. Conclusions

After years of relative obscurity, there have been rapid recent advances in understanding the biology of BAIs and other adhesion-GPCRs. These molecules are intriguing because they tend to have multiple ligand binding domains that suggest that they are signal integrators, recognize large, complex substrates, and/or detect coincidences. The complexities added by NTF swapping, signaling by both GPCR-dependent and -independent modes, splice variants, and potential formation of higher level complexes are only beginning to be understood in a functional context. These complexities lend themselves to neuronal and synaptic function, given the role that these cells and structures play in storing and processing information. BAIs in particular are demonstrating key roles in synaptic function, though they play other roles in and out of the brain as well. A full appreciation of BAI function will require the identification of all BAI ligands, complete elucidation of BAI expression patterns and localization, identification of all binding partners and modes of signaling, and dynamic measurements of these properties. These are exciting challenges that hold great promise for increasing our understanding of synaptic function, as well as treating synaptic dysfunction.
